# Sub-inhibitory concentrations of ceftriaxone induce morphological alterations and PIA-independent biofilm formation in *Staphylococcus aureus*

**DOI:** 10.1007/s42770-023-01177-x

**Published:** 2023-11-18

**Authors:** Ahmed Azzam, Riham M. Shawky, Taghrid S. El-Mahdy

**Affiliations:** 1 Department of Microbiology and Immunology, Faculty of Pharmacy, Helwan University, Ain Helwan, Cairo, Egypt; 2 Department of Microbiology and Immunology, Faculty of Pharmacy, Modern University for Technology and Information (MTI), Cairo, Egypt

**Keywords:** Ampicillin, Ceftriaxone, Gentamicin, Norfloxacin, S*taphylococcus aureus*, Biofilm, Morphology

## Abstract

The exposure of bacteria to sub-inhibitory concentrations of antibiotics is of biological significance since it can occur in vivo under many circumstances, including low-dose treatment, poor adherence to a regimen, poor drug penetration, drug-drug interactions, and antibiotic resistance of the pathogen. In this study, we investigated the effects of subinhibitory concentrations of four antibiotics: ampicillin, ceftriaxone, gentamicin, and norfloxacin, which are commonly used in clinical settings and on cell morphology and biofilm formation in *Staphylococcus aureus* as one of the leading causes of nosocomial and biofilm-associated infections. Nine clinical *S. aureus* biofilm-producing isolates and two known biofilm-producing reference strains, *S. aureus* ATCC 29213 and *S. aureus* ATCC 6538, were used in this study. Sub-MICs of beta-lactam antibiotics (ampicillin and ceftriaxone) significantly induced biofilm formation in *S. aureus* ATCC 29213 and *S. aureus* ATCC 6538 and in six clinical isolates out of the nine selected isolates when compared with the antibiotic-free control group (*P* < *0.05*), with an approximately 2- to 2.5-fold increase. Gentamicin and norfloxacin induced biofilms in *S. aureus* ATCC 29213 and *S. aureus* ATCC 6538, while gentamicin and norfloxacin induced biofilms only in three and two of the nine tested isolates, respectively (*P* < *0.05*). The chemical nature of the biofilm matrix produced by half the MIC of ceftriaxone in the six isolates that showed increased biofilm was all non-polysaccharide in composition (PIA-independent). Gene expression of biofilm-encoding genes *atl* and *sarA* in biofilms of the two tested strains (*S. aureus* ATCC 6538) and clinical strain (*S. aureus* 16) showed a significant upregulation after exposure to half MIC of ceftriaxone. Additionally, the bacterial cell morphological changes in planktonic cells caused by half MIC of ceftriaxone were evaluated by scanning electron microscopy, which demonstrated a significant cell enlargement when compared with the antibiotic-free control (*P* < *0.05*), and some deformed cells were also noticed. In *S. aureus* clinical isolates, sub-MICs of ampicillin, ceftriaxone, gentamicin, and norfloxacin may stimulate substantial production of biofilm, which could have important clinical significance and make infection treatment challenges. Further, in vivo research is needed to fully comprehend how sub-MIC of antibiotics can affect biofilm formation in clinical settings. Additionally, more research is required to reveal the clinical implications of the morphological alterations in *S. aureus* brought on by exposure to ceftriaxone at concentrations below its MIC.

**Graphical Abstract:**

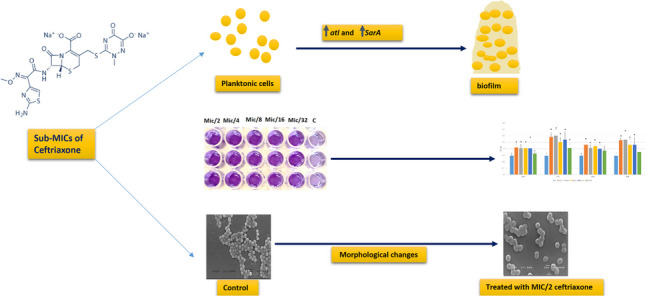

## Introduction

*Staphylococcus aureus* (S*. aureus*) is one of the leading causes of biofilm-related infections [1]. It is known to form biofilms on medical devices such as catheters, implants, and prosthetic joints, leading to chronic infections that are difficult to treat [1]. During antibiotic treatment, bacteria may be exposed to the sub-inhibitory concentrations (sub-MICs) of antibiotics, which were defined by Andersson and Hughes as “the concentration that allows susceptible strains to continue to grow, which sometimes results in a reduced growth rate compared with the growth rate that is observed in the absence of the drug” [2]. Such exposure can occur for a variety of reasons, including incorrect dosages, poor adherence to a regimen, poor penetration, drug-drug interactions, and antibiotic resistance of the bacteria [3]. The effects of subinhibitory levels of antibiotics on *S. aureus* may include potential effects on cell morphology, expression of virulence factors, adhesion and invasion of the organism to host, biofilm formation, and small-colony variant (SCV) production [3].

Biofilms are a group of microbial cells that attach to the surface of living or inanimate objects and coat themselves in a self-produced extracellular matrix. The matrix composition is known to differ between and within species, but it mainly consists of polysaccharides, DNA, and proteins [4].

Biofilm formation in *S. aureus* is regulated by *icaADBC*-dependent and -independent pathways [5]. In the *ica*-dependent biofilm, the polysaccharide intercellular adhesin (PIA), which facilitates bacterial intercellular adhesion and the development of biofilms, is produced by the genes of the *ica* operon [5]. In the *ica*-independent or PIA-independent biofilm, specific proteins or extracellular DNA (eDNA) can substitute for PIA in cell–cell adhesion [5, 6]. It was reported that methicillin-sensitive *S. aureus* (MSSA) strains frequently develop a PIA-dependent biofilm. In contrast, eDNA and proteins dominated the biofilm matrix of methicillin-resistant *S. aureus* (MRSA) [7].

Several aspects of biofilm formation make it a clinically relevant process: First, antimicrobial agents tend to have a much weaker effect on bacteria embedded in biofilms compared with those in a planktonic state, which allows bacteria to resist antibacterial agents. Second, biofilm may be a persistent source of infection. Third, they may allow the exchange of resistance plasmids [8, 9].

A previous study demonstrated that sub-MICs of ampicillin significantly induce biofilm formation in some strains of *S. aureus* [10]. However, there are no studies that assess the sublethal effect of ceftriaxone and gentamicin on *S. aureus*. Since there are a number of in vivo circumstances where concentrations of these antibiotics may be at subinhibitory levels, the objective of this study was to measure biofilm formation by clinically isolated *S. aureus* in response to sub-MICs of four antibiotics (ampicillin, ceftriaxone, gentamicin, and norfloxacin) that are commonly used in clinical settings by using the crystal violet method and quantitative PCR (qPCR). We further addressed the nature of *S. aureus* biofilm matrix composition as a result of such antibiotic sub-MIC exposure. We also aimed to characterize the potential morphological alterations in *S. aureus* caused by sub-MIC of ceftriaxone.

## Materials and methods

### Bacterial strains

A total of forty (40) clinical isolates, preliminary identified as *Staphylococcus* species, were obtained from different infected patients admitted to Kasr El-Aini Teaching Hospital from July until October 2021. The isolates were obtained as follows: two isolates from sputum, two from pleural aspirates, 17 from blood, six from pus, one from semen, one from vaginal swab, one from urine, nine from wounds, and one from ascetic fluid. *S. aureus* ATCC 29213 (a reference strain for broth microdilution technique and a known biofilm producer) and *S. aureus* ATCC 6538 were kindly provided by the Microbiology Department, Faculty of Agriculture, Cairo University, Giza, Egypt, whereas *S. aureus* ATCC 25923 (a reference strain for disc diffusion technique) was obtained from the Cairo Microbiological Resources Center (Cairo MIRCEN).

### Phenotypic and molecular identification of *S. aureus* isolates

Isolates were submitted to Gram staining, catalase, and tube coagulase tests. In addition, species confirmation was made using PCR according to the method developed by Martineau et al. [11]*.*

### MRSA identification

Cefoxitin disc diffusion method was carried out according to Clinical and Laboratory Standards Institute (CLSI) procedures on Mueller–Hinton agar by using a 30 µg cefoxitin disc (HiMedia, Mumbai, India). An inhibition zone diameter of ≤ 21 mm was reported as methicillin-resistant, and a diameter of ≥ 22 mm was considered methicillin-sensitive. MRSA isolates were further identified by detection of the *mecA* gene [12].

### Preparation of antimicrobial agents

Ampicillin, ceftriaxone, norfloxacin, and gentamicin standards were generously obtained from the Egyptian Pharmaceutical International Company (EPICO), Egypt. Antibiotic stock solutions were prepared according to “CLSI M07 2012 methods for dilution antimicrobial susceptibility testing for bacteria that grow aerobically” and stored at − 20 °C. The concentration of all stock solutions was 5120 µg/mL.

### Screening of biofilm production by clinical isolates

The biofilm formation assays were performed by the crystal violet staining method using sterile 96-well polystyrene plates using the method adopted by Yang et al. [13]. Confirmed isolates of *S. aureus* were grown on Tryptic soya agar (TSA, Oxoid, Basingstoke, UK) at 37 °C and incubated overnight. Five-ml tryptic soya broth (TSB; Condalab, Madrid, Spain) tubes were used to inoculate the overnight TSA, and the concentrations were adjusted to 1.0 × 10^7^ CFU/ml. The bacterial suspension was transferred to 96 flat-bottomed, sterile polystyrene microplates (100 µL/well) at a concentration of 1.0 × 10^7^ CFU/mL. A medium lacking bacteria was used as blank control. The contents of each well were removed after 24 h of incubation at 37 °C without shaking, and the wells were gently washed twice with sterile distilled water. After air-drying, the plates were stained with 120 µL of 1% w/v crystal violet solution (HiMedia, India). The crystal violet was removed after 15 to 20 min, and 300 µL of distilled water was used to rinse the wells. To dissolve dye that was bound to bacteria, 150 µL of 33% glacial acetic acid was used for 10 min. The absorbance in each well was measured with a spectrophotometer at 630 nm (ELX808, BioTeK, USA). The strains were classified into four different categories according to their biofilm-forming ability depending on their Optical density cut-off value “ODc” (ODc = OD average of blank well + (3 × Standard deviation of blank well)) [13]. The bacterial biofilm categories are as follows: not able to form a biofilm (2 ODc < OD < 4 ODc), moderately able to form a biofilm (4 ODc < OD < 6 ODc), and strongly able to form a biofilm (6 ODc < OD). The tests were performed in triplicate at three independent repeats.

### MIC determination

Minimum inhibitory concentrations (MICs) for ampicillin, ceftriaxone, norfloxacin, and gentamicin were determined by the broth microdilution method, according to CLSI Performance Standards for Antimicrobial Susceptibility Testing, 2021.

### Effect of sub-MICs of antibiotics on biofilm formation with crystal violet staining

Isolates were exposed to previously mentioned antibiotics 1/2 MIC to 1/32 MIC and supplemented with TSB at 37 °C for 24 h. Wells lacking drugs and lacking bacteria in the same medium served as the negative control and blank control, respectively. After incubation, biofilm formation was quantified by crystal violet staining as mentioned above. The tests were performed in triplicate at three independent repeats.

### Biofilm detachment assay

The chemical nature of the biofilm matrix produced by 1/2 MIC of ceftriaxone was determined by degradation with 40 mM sodium metaperiodate (Sisco Research Laboratories Pvt. Ltd., Mumbai, India), 100 µg/ml proteinase K (Sigma, USA) solutions in 0.1 M phosphate-buffered saline (PBS) (pH 7.0), and DNase I (Thermo Fisher Scientific, USA) (100 µg/ml in 150 mM NaCl and 1 mM CaCl2) in a test resembling the microtiter plate biofilm formation assay [14]. Microtiter plates were seeded with 100 µL per well of the bacterial suspensions exposed to 1/2 MIC of ceftriaxone and incubated at 37 °C for 24 h. Subsequently, the cultures were removed and the wells were washed with purified water. Degrading agents and PBS (control), 100 µL per well, were put in triplicate wells, and the plates were incubated for 2 h at 35 °C. Following this, the wells were washed twice with distilled water, and the next steps were developed as described in the microtiter plate biofilm assay. A reduction of over 50% in the OD average, when compared to the control, of wells treated with degrading agents indicated the chemical nature of the biofilm [15]. The tests were performed in triplicate at three independent times.

### Morphological observations of planktonic *S. aureus* exposed to half the subMIC of ceftriaxone by scanning electron microscopy (SEM)

*S. aureus* isolates were treated with ceftriaxone at 1/2 the MIC for 24 h at 37 °C. After incubation, the bacterial suspensions were centrifuged, and the bacterial cells were fixed in 2.5% glutaraldehyde in PBS (pH 7.4) for 1 h at room temperature. The fixed samples were then washed three times with PBS for 10 min and dehydrated for 30 min in a graded ethanol series. After critical-point drying, the samples were mounted on stubs, coated with gold, and observed by scanning electron microscopy (SEM; JEOL GM 5200 microscope, JEOL, Ltd., Akishima, Japan). Nine individual bacteria were randomly selected in each microscopic field for diameter length measurements.

### Biofilm-related genes evaluation by real-time polymerase chain reaction

One clinical strain and *S. aureus* ATCC 6538 were selected to evaluate the biofilm-related genes *icaR*, *sarA*, *fnbA*, and *atl*. *16S rRNA* gene was used as an endogenous control of reactions. The primer sequences are listed in Table 1. Fifty microliters of the overnight cultures of *S. aureus* strains were diluted in TSB to 1 × 10^7^ CFU/ml and then added into a 96-well flat bottom plate together with 50 µL of 1/2 MIC ceftriaxone and incubated overnight. The mixture of TSB with ceftriaxone at 1/2 MIC and *S. aureus* cultures was transferred into Eppendorf tubes. The mixtures were then centrifuged at 5000 g for 10 min. After the supernatants were removed, the pellets were kept at − 80 °C. The pellets were lysed using 0.1-ml of Tris–EDTA buffer supplemented with 0.2 mg/ml of lysostaphin (Sigma-Aldrich) and then incubated at 37 °C for 30 min. RNA was extracted from the lysed cells using the GENEzol™ reagent (Geneaid, Taiwan) according to the manufacturer’s instructions. The concentration of total RNA was measured using a NanoDrop 2000 spectrophotometer (Thermo Fisher Scientific, USA). RNA samples that had a 260/280 ratio between 2.0 and 2.2 were reverse-transcribed with TOPreal™ One-Step RT qPCR Kit (Enzynomics, Korea) as indicated by the manufacturer’s instructions. Each PCR reaction tube contained 20 µL reaction mixtures consisting of the following: 1 µL TOPreal TM One-Step RT qPCR Enzyme Mix, 10 µL TOPreal TM One-Step RT qPCR Reaction Mix, 2 µL of RNA extract, 1 µL of each primer( 10 pmol/µl) and 5 µL RNAase-free water. The reacting condition was set as a one-step method as follows: synthesize cDNA at 50 °C for 30 min, initial denaturation at 95 °C for 10 min, followed by 40 cycles of denaturation at 95 °C for 5 s, and annealing at 60 °C for 30 s. All samples were carried out in duplicate, and three independent experiments were performed. Expression levels of the genes were normalized to *16S rRNA*. The changes in each transcript were determined by the 2^−ΔΔT^ method when compared to drug-free cells.

**Table 1:** Oligonucleotide primers used in the quantitative real-time PCR analysis

Gene name	Primer	Sequence 5′—3′	Reference
*icaR*	*icaR-F*	ATCTAATACGCCTGAGGA	[16]
	*icaR-R*	TTCTTCCACTGCTCCAA	
*sarA*	*sarA-F*	TCTTGTTAATGCACAACAACGTAA	[17]
	*sarA-R*	TGTTTGCTTCAGTGATTCGTTT	
*fnbA*	*fnbA-F*	GAAGAGCATGGTCAAGCACA	
	*fnbA-R*	ACGTCATAATTCCCGTGACC	
*atl*	*atl-F*	ATAACCGCACTGGTTGGGTA	
	*atl-R*	TTGGCAGCTGATGTAGTTGG	
*16S rRNA*	*16S rRNA-F*	GAGGGTCATTGGAAACTGGA	
	*16S rRNA-R*	CATTTCACCGCTACACATGG	

### Statistical analysis

The experimental data were analyzed using a one-way analysis of variance (ANOVA) by SPSS 22.0, and the statistical results are reported as a mean ± standard deviation. Pairwise comparisons with differences of *P* ≤ 0.05 were considered statistically significant; *P* ≤ 0.01 was considered extremely significant. The independent sample *t*-test was used by comparing the ΔCt for treatment and control for gene expression experiments.

## Results

### Identification

**Fig. 1:**
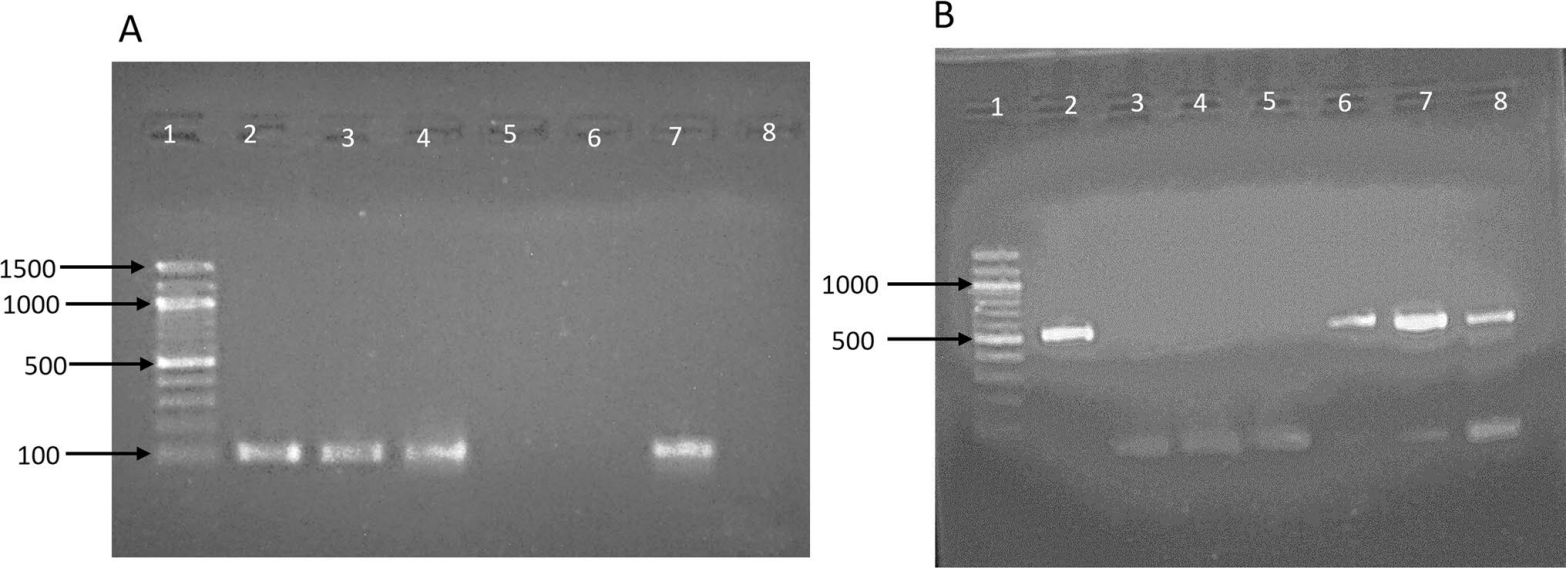
Molecular identification and characterization of *S. aureus*. **A** PCR amplification of *S. aureus* specific fragment “SA442” (amplicon 108 bp). Lane 1: DNA ladder, lane 2: positive control, lane: 3 to 7 clinical isolates that were mannitol fermenters on MSA but negative coagulase, lane 8 negative control. **B** PCR amplification of *mecA* gene (amplicon 530 bp) Lane 1: DNA ladder, lane 2: positive control, lane: 3 to 8 *S. aureus* isolates

Forty (40) clinical isolates preliminary identified as *Staphylococcus* species were obtained from different infection sources. All were slide catalases and gram-positive cocci arranged in clusters. Thirty-seven isolates (37) were mannitol fermenters on MSA, and of them, 32 were positive for the tube coagulase test. Out of the five mannitol fermenters and coagulase negative, PCR confirmation revealed three isolates to be *Staphylococcus aureus* (Fig. 1). Thirty-two (32) of the 35 confirmed *S. aureus* isolates were identified as MRSA and three as MSSA by cefoxitin disc diffusion and PCR (Fig. 1).

### Screening of biofilm production

Table 2 shows the biofilm category with its corresponding optical density range. Biofilm quantification using crystal violet staining demonstrated that 19 of 35 (54.28%) were biofilm producers. Eleven isolates were categorized as weak, five as moderate, and three as strong biofilm producers. The remaining isolates were classified as non-producers because their optical densities ranged from 0.05 to 0.108. Nine clinical isolates were chosen at random for further investigation (three of each weak, moderate, and strong biofilm producer). The susceptibilities of the chosen isolates and their biofilm production are shown in Table 3.

**Table 2:** The biofilm category with their corresponding optical density range

Biofilm category*	O.D range (630 nm)	Number of isolates
Non producer	0.054–0.108	16
Weak	0.109–0.216	11
Moderate	0.217–0.324	5
Strong	> 0.324	3

*The average Optical density of the blank = 0.051 and standard deviation = 0.001

Abbreviation: *O.D* optical density

**Table 3:** The susceptibilities of the chosen isolates and their biofilm category

Clinical isolate /reference strain	MIC µg/mL				Biofilm category	Source of isolation
	Ampicillin	Ceftriaxone	Gentamicin	Norfloxacin		
*S. aureus* ATCC 6538	0.125 (S)	1 (S)	0.25 (S)	0.5 (S)	Moderate	_
*S. aureus* ATCC 29213	4 (S)	2 (S)	1 (S)	1 (S)	Weak	_
SA 11	128 (R)	64 (R)	8 (I)	64 (R)	Weak	Urine
SA 22	64 (R)	64 (R)	1 (S)	64 (R)	Moderate	Sputum
SA 33	> 512 (R)	128 (R)	2 (S)	8 (I)	Weak	Pleural aspirate
SA 25	256 (R)	256 (R)	8 (I)	8 (I)	Strong	Blood
SA 35	> 512 (R)	128 (R)	4 (S)	16 (R)	Weak	wound
SA 16	32 (R)	4 (S)	1 (S)	1 (S)	Strong	Urine
SA 6	256 (R)	64 (R)	8 (I)	32 (R)	Moderate	wound
SA 19	> 512 (R)	64 (R)	2 (S)	32(R)	Moderate	wound
SA 28	256 (R)	128(R)	64 (R)	64 (R)	Strong	Blood

Abbreviation:* S* sensitive, *I* intermediate, *R* resistant according to CLSI guidelines, *SA*: *Staphylococcus aureus*

### Effect of sub-MICs of antibiotics on biofilm formation

**Fig. 2:**
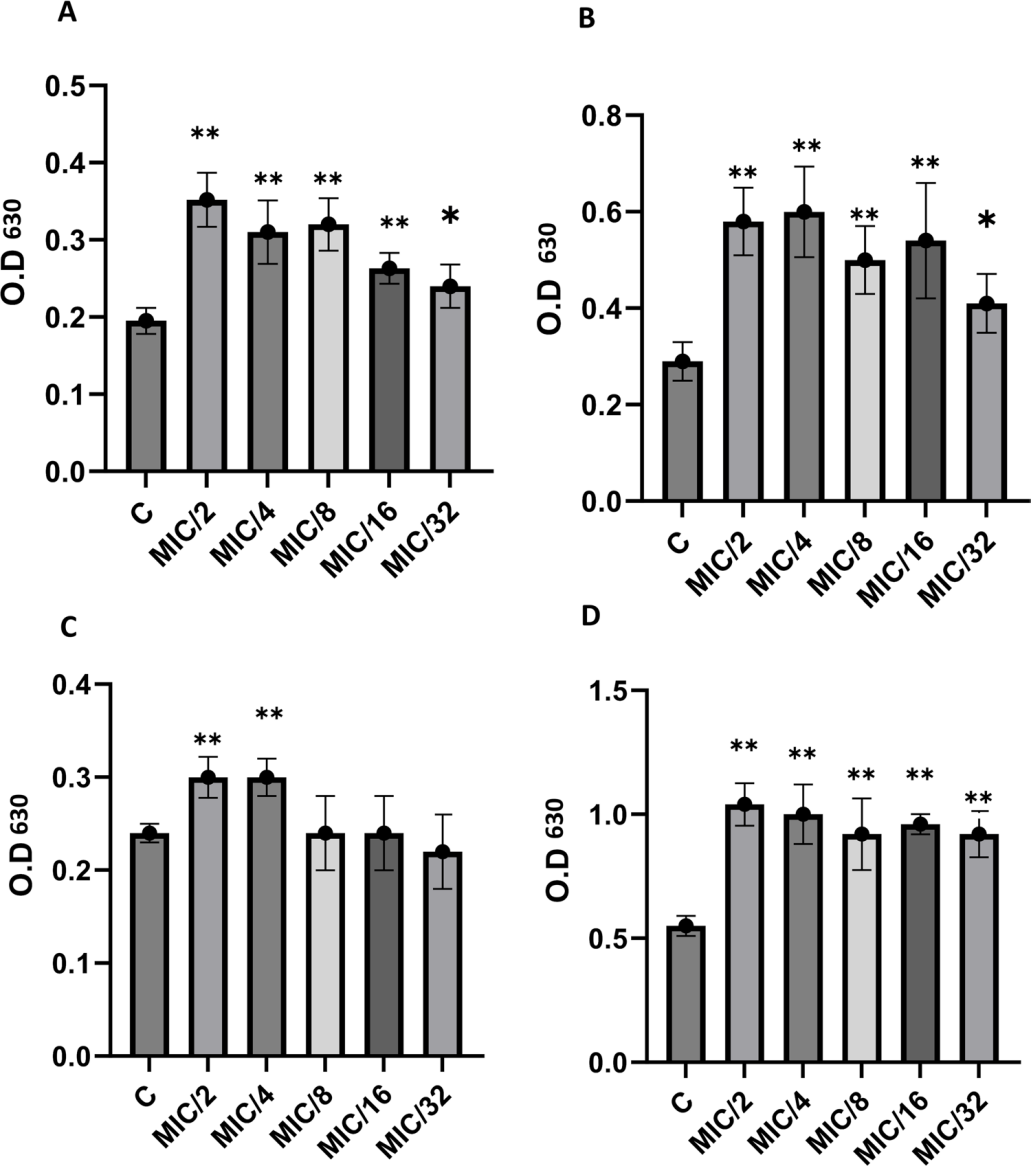
Effects of ceftriaxone sub-MICs (MIC/2 to MIC/32) on *S. aureus* biofilm formation with crystal violet staining at a wavelength of 630 nm (OD630). “C” refers to the untreated control. The data shown are representative of three independent experiments, and bars indicate the mean values ± standard deviation. **A** *S. aureus* ATCC 29213. **B** *S. aureus* ATCC 6538. **C** *S. aureus* 16. **D** *S. aureus* 22. An asterisk “*” indicates statistical significance where the *P-*value is less than 0.05. The notation “**” denotes a high level of statistical significance, indicating that the corresponding *P*-value is less than 0.01

Two known biofilm formers (*S. aureus* ATCC 29213 and *S. aureus* ATCC 6538) and nine clinical isolates (three of each weak, moderate, and strong biofilm producer) were chosen to investigate the effect of sub-MICs of antibiotics on biofilm formation. Strains were exposed to sub-MICs (1/2 MIC to 1/32 MIC) of ampicillin, ceftriaxone, gentamicin, and norfloxacin for 24 h, and biofilm formation was compared with that of strains cultured without antibiotics. Out of the nine clinical isolates, three clinical isolates that were weak biofilm producers were not significantly affected by sub-MIC antibiotics (SA 11, 33, and 35). However, sub-MICs of beta-lactam antibiotics (ampicillin and ceftriaxone) significantly induced biofilm formation for the other six isolates and *S. aureus* ATCC 29213 and *S. aureus* ATCC 6538 when compared with the antibiotic-free control group (*P* < 0.05) at all sub-MICs tested (1/2 MIC to 1/32 MIC), suggesting a strain-dependent behavior. Ampicillin and ceftriaxone were able to induce biofilms 2- to 2.5-fold compared with the antibiotic-free control group; in most cases, the biofilm biomass induced by sub-MICs of ceftriaxone was slightly higher than that of ampicillin. When compared to control, gentamicin and norfloxacin statistically induced biofilms in *S. aureus* ATCC 29213 and *S. aureus* ATCC 6538, as well as in three (SA 22, 6, and 19) and two (SA 22 and 6) of the nine tested isolates, respectively (*P* < 0.05). Figure 2 illustrates the graphical depiction of the impact of ceftriaxone sub-MIC on the formation of *S. aureus* biofilm. The average optical densities with standard deviations at various sub-MICs (1/2 MIC to 1/32 MIC) of the four antibiotics examined are shown in Table 4.

**Table 4:** The average optical densities after crystal violet assay at 1/2 MIC to 1/32 MIC of the four tested antibiotics

Average O.D. _630_ (with its SD) of untreated *S. aureus* strains	Tested antibiotic	Average O.D. _630_ (with its SD) at 1/2 MIC to 1/32 MIC				
		MIC/2	MIC/4	MIC/8	MIC/16	MIC/32
ATCC 29213 O.D = 0.195 (0.0172)	AMP	0.385 (0.0687)**	0.338 (0.043)**	0.333 (0.065)**	0.315 (0.04)**	0.3153 (0.04)**
	CEF	0.352 (0.035)**	0.31 (0.041)**	0.32 (0.034)**	0.263 (0.02)**	0.24 (0.028)*
	GEN	0.31 (0.025)**	0.285 (0.044)**	0.27 (0.046)**	0.29 (0.024)**	0.256 (0.032)**
	NOR	0.33 (0.056)**	0.3 (0.062)*	0.26 (0.073)	0.25 (0.063)	0.245 (0.05)
ATCC 6538 O.D = 0.29 (0.04)	AMP	0.418 (0.053)*	0.407 (0.0596)*	0.409 (0.0816)*	0.405 (0.125)*	0.324 (0.056)
	CEF	0.58 (0.07)**	0.6 (0.094)**	0.5 (0.07)**	0.54 (0.12)**	0.41 (0.061)*
	GEN	0.46 (0.047)**	0.41 (0.044)**	0.44 (0.05)**	0.4 (0.063)**	0.37 (0.078)
	NOR	0.53 (0.031)**	0.54 (0.12)**	0.46 (0.085)**	0.46 (0.12)*	0.35 (0.049)
SA 11 O.D = 0.124 (0.0092)	AMP	0.124 (0.0087)	0.119 (0.01)	0.123 (0.005)	0.115 (0.0097)	0.115 (0.009)
	CEF	0.132 (0.009)	0.121 (0.0125)	0.113 (0.0162)	0.12 (0.0137)	0.117 (0.012)
	GEN	0.137 (0.0174)	0.141 (0.01)	0.137 (0.015)	0.123 (0.0131)	0.118 (0.0092)
	NOR	0.139 (0.0176)	0.136 (0.0082)	0.133 (0.0163)	0.116 (0.0124)	0.116 (0.0129)
SA 22 O.D = 0.24 (0.01)	AMP	0.3 (0.04)**	0.29 (0.026)*	0.266 (0.023)	0.256 (0.042)	0.22 (0.04)
	CEF	0.3 (0.022)**	0.3 (0.02)**	0.24 (0.04)	0.24 (0.04)	0.22 (0.04)
	GEN	0.52 (0.052)**	0.5 (0.05)**	0.5 (0.07)**	0.39 (0.064)**	0.36 (0.054)**
	NOR	0.313 (0.036)**	0.316 (0.051)**	0.321 (0.056)**	0.34 (0.034)**	0.32 (0.057)**
SA 33 O.D = 0.178 (0.016)	AMP	0.17 (0.012)	0.16 (0.014)	0.168 (0.006)	0.161 (0.015)	0.169 (0.0196)
	CEF	0.187 (0.0151)	0.165 (0.016)	0.163 (0.026)	0.16 (0.0183)	0.164 (0.025)
	GEN	0.206 (0.026)	0.2 (0.015)	0.193 (0.023)	0.168 (0.018)	0.174 (0.0175)
	NOR	0.2 (0.028)	0.2 (0.012)	0.193 (0.023)	0.16 (0.017)	0.16 (0.0234)
SA 25 O.D = 0.4 (0.015)	AMP	0.64 (0.078)**	0.67 (0.089)**	0.646 (0.0679)**	0.66 (0.097)**	0.67 (0.08)**
	CEF	0.9 (0.07)**	0.79 (0.07)**	0.75 (0.07)**	0.73 (0.063)**	0.72 (0.079)**
	GEN	0.41 (0.07)	0.39 (0.038)	0.42 (0.056)	0.43 (0.044)	0.4 (0.05)
	NOR	0.379 (0.024)	0.38 (0.038)	0.384 (0.043)	0.37 (0.037)	0.368 (0.04)
SA 35 O.D = 0.167 (0.019)	AMP	0.2 (0.02)	0.204 (0.028)	0.175 (0.021)	0.191 (0.0284)	0.178 (0.02)
	CEF	0.164 (0.021)	0.172 (0.05)	0.183 (0.049)	0.185 (0.015)	0.191 (0.025)
	GEN	0.194 (0.042)	0.19 (0.021)	0.178 (0.03)	0.174 (0.023)	0.176 (0.029)
	NOR	0.169 (0.016)	0.168 (0.018)	0.17 (0.019)	0.175 (0.0197)	0.16 (0.025)
SA 16 O.D = 0.55 (0.041)	AMP	0.89 (0.093)**	0.92 (0.141)**	0.879 (0.107)**	0.84 (0.071)**	0.77 (0.079)**
	CEF	1.04 (0.086)**	1 (0.12)**	0.92 (0.144)**	0.96 (0.04)**	0.92 (0.093)**
	GEN	0.542 (0.067)	0.554 (0.062)	0.55 (0.04)	0.53 (0.0532)	0.56 (0.034)
	NOR	0.57 (0.049)	0.55 (0.06)	0.571 (0.07)	0.549 (0.0387)	0.566 (0.03)
SA 6 O.D = 0.236 (0.019)	AMP	0.322 (0.039)**	0.275 (0.008)*	0.28 (0.021)**	0.281 (0.035)**	0.236 (0.0187)
	CEF	0.353 (0.028)**	0.319 (0.018)**	0.306 (0.054)**	0.286 (0.0411)*	0.293 (0.029)*
	GEN	0.343 (0.035)**	0.334 (0.0376)**	0.274 (0.054)	0.287 (0.034)	0.291 (0.045)
	NOR	0.36 (0.032)**	0.322 (0.03)**	0.297 (0.04)**	0.272 (0.024)*	0.287 (0.0346)*
SA 19 O.D = 0.255 (0.0186)	AMP	0.32 (0.031)**	0.303 (0.0238)*	0.324 (0.038)**	0.301 (0.025)*	0.307 (0.034)*
	CEF	0.45 (0.054)**	0.364 (0.031)**	0.361 (0.04)**	0.335 (0.0463)**	0.32 (0.059)*
	GEN	0.414 (0.076)**	0.343 (0.044)**	0.351 (0.0412)**	0.266 (0.047)	0.3 (0.032)
	NOR	0.246 (0.032)	0.236 (0.012)	0.258 (0.047)	0.216 (0.024)	0.25 (0.052)
SA 28 O.D = 0.351 (0.016)	AMP	0.83 (0.069)**	0.67 (0.088)**	0.635 (0.072)**	0.586 (0.04)**	0.479 (0.058)**
	CEF	0.83 (0.093)**	0.75 (0.04)**	0.62 (0.063)**	0.475 (0.087)**	0.41 (0.041)
	GEN	0.352 (0.0269)	0.324 (0.0363)	0.34 (0.0597)	0.297 (0.066)	0.348 (0.0336)
	NOR	0.353 (0.039)	0.358 (0.0325)	0.324 (0.0451)	0.336 (0.053)	0.32 (0.06)

Abbreviations: *AMP* ampicillin, *CEF* ceftriaxone, *GEN* gentamicin, *NOR* norfloxacin, *O.D*: optical density, *SD* standard deviation, *P < 0.05, ** *P* < 0.01

### Determination of biofilm chemical nature

The chemical composition of the biofilm matrix was investigated in six isolates that produced biofilms in response to sub-MICs of ceftriaxone and reference strains on the basis of the degree of degradation after treatment with metaperiodate, proteinase K, and DNAse. All biofilms induced were non-polysaccharides (PIA-independent) in composition: Protein (SA 19 and 28) and eDNA (SA 22, 25 and 16). The induced biofilm was not dispersed by any of the tested agents in one isolate (SA 6), while *S. aureus* ATCC 29213 and *S. aureus* 6538 showed a polysaccharide and proteinaceous matrix, respectively.

### Morphological observations of planktonic *S. aureus* exposed to half the subMIC of ceftriaxone by scanning electron microscopy (SEM)

**Fig. 3:**
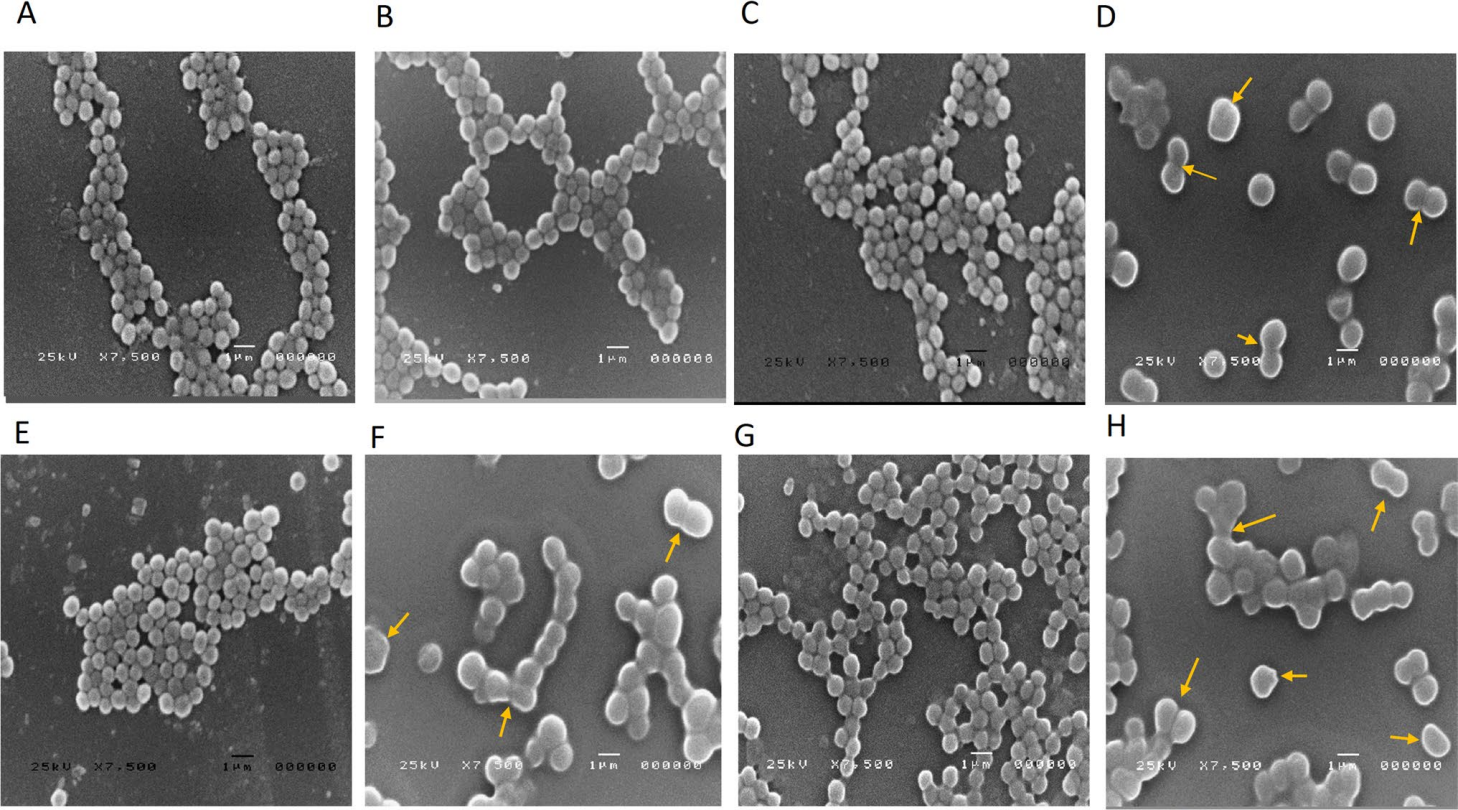
Morphological changes in planktonic cells of *S. aureus* caused by 1/2 MIC of ceftriaxone. Scale bars = 1 µm. The yellow arrows point at deformed *S. aureus* cells: irregular cell shapes and abnormally fused cells. **A** and **B** Untreated and treated *S. aureus* 6538, respectively. **C** and **D** Untreated and treated *S. aureus* 35, respectively. **E** and **F** Untreated and treated *S. aureus* 22, respectively. **G** and **H** Untreated and treated *S. aureus* 16, respectively

**Fig. 4:**
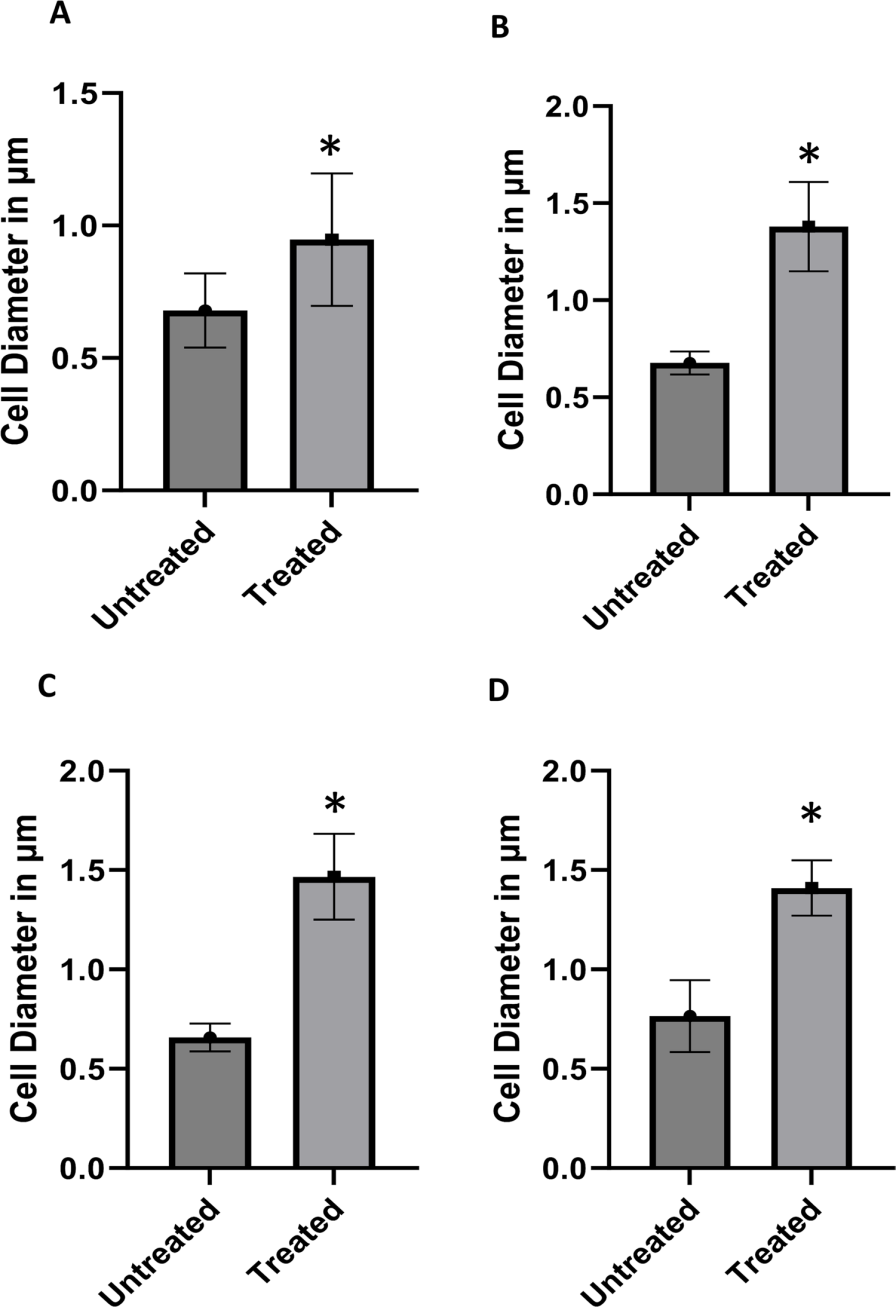
Morphological changes in planktonic cells of *S. aureus* caused by 1/2 MIC of ceftriaxone. Nine individual bacteria were randomly selected in each microscopic field for treated and untreated cells. The diameter length was measured using ImageJ software. **A** *S. aureus* 6538. **B**, **C**, and **D** Three clinical isolates of *S. aureus* 35, 22, and 16, respectively. An asterisk “*” indicates statistical significance where the *P-*value is less than 0.05

The bacterial cell morphological changes in planktonic cells by 1/2 MIC of ceftriaxone are shown in Fig. 3. *S. aureus* ATCC 6538 and three clinical isolates (one weak, one moderate, and one strong biofilm producer) were selected. Two main types of damage were observed: cell enlargement and deformed cells. The average bacterial cell diameter in the control group of the tested isolates was approximately 0.6 to 0.7 µm. All of the tested isolates showed a statistically significant increase in cell diameter when compared with the control, which reached a twofold or greater increase (Fig. 4) for the clinical isolates tested (*S. aureus* 22, 16, and 35).

### Expression of biofilm-related genes

**Fig. 5:**
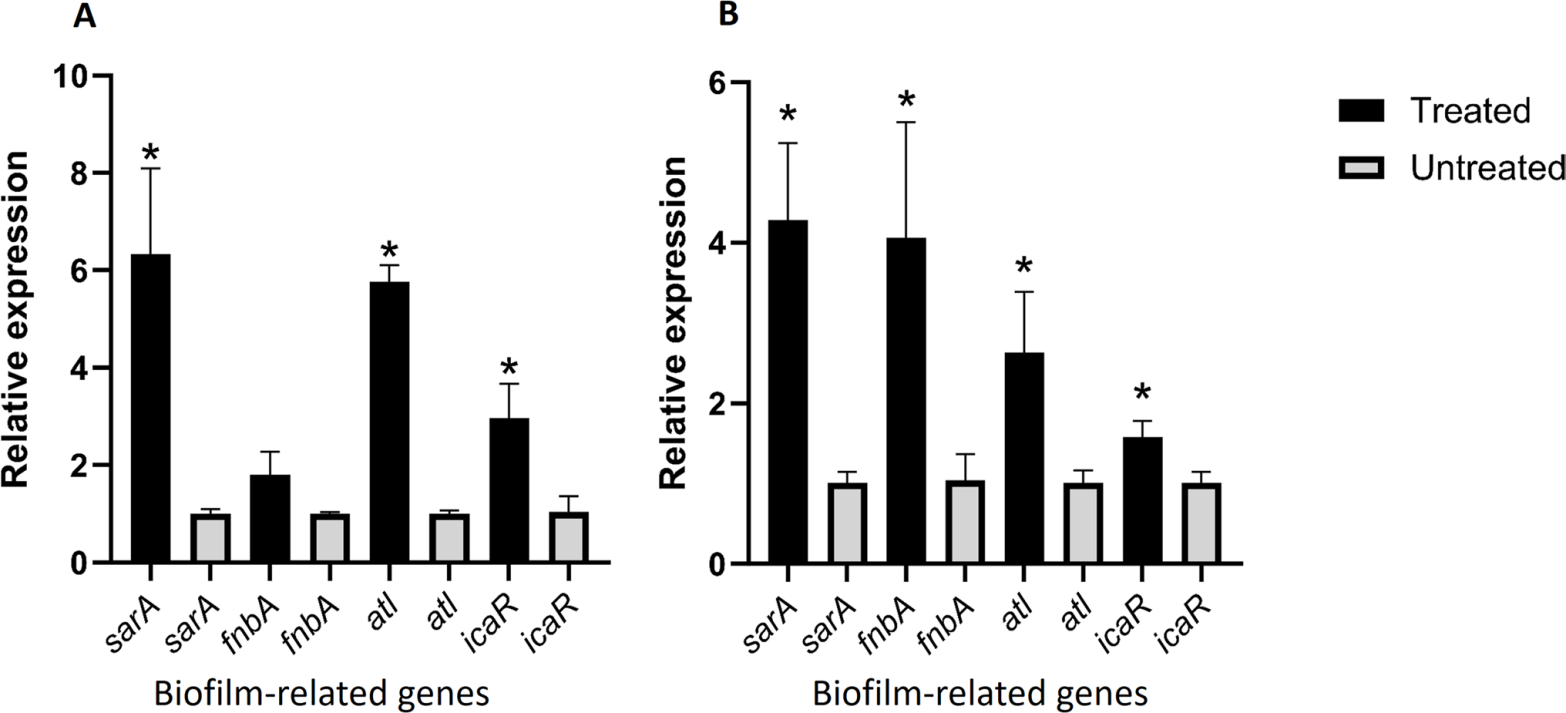
Effects of MIC/2 of ceftriaxone on *S. aureus* biofilm-related genes relative expression compared with untreated control. **A** *S. aureus 16*. **B** *S. aureus* 6538. Quantification of the transcript was obtained using the 2^−ΔΔCt^ method versus the untreated control. All data are shown as the mean ± SD from three independent experiments. * indicates statistical significance (*P* < 0.05)

To determine whether the influence of antibiotics was manifested at the transcriptional level, total RNA was isolated from *S. aureus* ATCC 6538 and *S. aureus* 16 following treatment at MIC/2 with ceftriaxone. The relative expression levels of the *icaR*, *sarA*, *fnbA*, and *atl* genes in *S. aureus* ATCC 6538 were significantly upregulated (*P* < 0.05) and reached fourfold upregulation in *sarA* and *fnbA*. In *S. aureus* 16, *sarA* and *atl* were about sixfold upregulated (*P* < 0.05). *icaR* was threefold upregulated (*P* < 0.05), whereas *fnbA* was not significantly upregulated. The transcription levels for all biofilm-related genes are shown in Fig. 5.

## Discussion

The exposure of bacteria to sub-inhibitory concentrations of antibiotics is of therapeutic significance since that exposure can occur under many circumstances. In this respect, sub-MICs of antibiotics are medically and biologically important. In our study, we revealed that sub-MICs of beta-lactam antibiotics (ampicillin and ceftriaxone) significantly induced biofilm formation in *S. aureus* ATCC 29213, *S. aureus* ATCC 6538, and most of the tested isolates. Gentamicin and norfloxacin statistically induced biofilms in *S. aureus* ATCC 29213, *S. aureus* ATCC 6538, and some of the tested clinical isolates. The chemical nature of the biofilm matrix produced by MIC/2 of ceftriaxone was all non-polysaccharides in composition (PIA-independent). Gene expression of *atl* and *sarA* in biofilms of two tested strains showed a significant upregulation after exposure to MIC/2 of ceftriaxone. In addition, a substantial cell enlargement in planktonic cells caused by MIC/2 of ceftriaxone was observed by SEM.

The effect of sub-MICs of antibiotics remains controversial, as some studies have shown that they can induce or decrease biofilm formation and can downregulate or upregulate gene expression in vitro in various gram-positive and gram-negative bacterial species [2]. Based on the strain tested and antibiotic used, antibiotics at sub-MICs may have different effects on *S. aureus* biofilm formation. Most antibiotics at sub-MICs inhibit the development of *S. aureus* biofilms, while certain antibiotics, such as oxacillin, ceftaroline, mupirocin, and rifampicin, promote staphylococcal biofilms [15, 18, 19].

Consistent with our findings, Kaplan et al. demonstrated that sub-MICs of β-lactam antibiotics (methicillin, ampicillin, amoxicillin, and cloxacillin) significantly induce biofilm formation in some strains of *S. aureus* via autolysin-dependent extracellular DNA release [10]. Another study demonstrated that the biofilm of *S. aureus* strain FAHGMU10071 showed a twofold increase in biomass and viability when treated with 1/4 MIC ampicillin for 8 h compared to the antibiotic-free control [20]. This increase in biofilm viability and biomass might be attributed to the upregulation of genes for the surface proteins *clfB*, *isdA*, and *sasG*, as well as genes (*cap5B and cap5C*) that support *S. aureus* adhesion [20]. To our knowledge, there are no studies that assess the sublethal effect of ceftriaxone and gentamicin on *Staphylococcus aureus* that can be compared with our findings, whereas only one study demonstrated that biofilm formation of *S. aureus* ATCC 25923 was enhanced under the sub-inhibitory stress of norfloxacin [21].

The composition of the biofilm extracellular matrix may vary depending on microbial species. For *S. aureus*, it consists of polysaccharides, extracellular DNA, and proteins. Most MRSA strains develop protein- and eDNA-based biofilms in the absence of antibiotics [7]. Using extracellular polymeric substances degradation assay, we found that sub-MIC of ceftriaxone induced biofilms of *S. aureus* via a PIA-independent pathway where eDNA and proteins dominated the matrix. According to Kaplan et al., sub-MICs of β-lactam antibiotics induced biofilm formation via the release of eDNA as evidenced by the following: first, the amount of eDNA in the biofilm matrix increased upon exposure to low-level methicillin; second, a strain carrying a mutation in the *atl* gene, which encodes the major *S. aureus* autolysin responsible for eDNA release, did not exhibit the biofilm induction phenotype; and third, the addition of exogenous DNase inhibited the biofilm induction phenotype [10]. By using confocal laser scanning microscopy, it was shown that the sublethal effects of mupirocin exposure promoted the formation of thick biofilms via eDNA [18]. The holin-like and antiholin-like proteins (encoded by the *cidA* gene), which regulate cell death and lysis during biofilm development, were mostly responsible for this impact. Additionally, mupirocin exposure did not cause the *cidA* mutant to produce thicker biofilms than the parent strain [22]. Mlynek et al. found that sub-MIC amoxicillin-induced biofilms of *S. aureus* with eDNA and proteinaceous adhesins dominated the matrix, while polysaccharide played a minor role in biofilm cohesion [23].

According to our finding, the relative expression levels of the *SarA* and *atl* genes were significantly upregulated (*P* < 0.05) in *S. aureus* strains treated with MIC/2 ceftriaxone compared with the untreated control. *atl*, the major *S. aureus* autolysin, increases biofilm formation by promoting cell lysis in a subset of the bacterial population, increasing eDNA accumulation and subsequent biofilm biomass [6]. The staphylococcal accessory regulator operon (*sarA*) encodes the SarA protein, which is a global transcriptional regulator that is also involved in the modulation of different virulence-related genes [6]. A number of studies indicated that the inactivation of *sarA* has a profound impact on *ica*-independent biofilm production in *S. aureus* [24–26]*.* The ability of *sarA* to enhance *ica*-independent biofilm production was likely to be associated primarily with its ability to repress proteases [25]. In addition, the *icaR* gene was significantly upregulated (*P* < 0.05). *IcaR* acts as a transcriptional repressor for the expression of the *icaADBC* operon that mediates PIA-dependent biofilm formation [5]. So it is also possible that ceftriaxone may induce PIA-independent biofilm formation via upregulation of *icaR*.

Our finding revealed that all of the tested isolates showed a statistically significant increase in cell diameter when compared with the control, which reached at least a twofold increase. Additionally, some deformed cells were noticed. Three main types of morphological changes in *S. aureus* were previously reported upon treatment with antibiotics at sub-MICs: cell morphology deformation, cell wall component changes, and cell wall breakdown [19, 27]. For instance, after exposure to the 1/2 to 1/8 MIC of dicloxacillin, cefodizime, cefotaxime, or ceftriaxone, MRSA USA300 and *S. aureus* ATCC 25923 cells were larger, damaged, had reduced adhesiveness, or had duplicate cells linked to one another [27]. In MRSA strains 06/1483 and 05/3291, the 1/2 MIC of ceftaroline can lead to cell wall damage and morphological changes in cells [19]. It is still unclear how and if deformed *S. aureus* caused by sub-MIC antibiotic exposure affects human immunity, bacterial pathogenicity, and antimicrobial sensitivity. These points require further investigation. On the same vein, sub-MICs of lincosamides and oxazolidinones were able to alter the *S. aureus* morphology allowing better opsonization and subsequent enhancement of phagocytosis [28]. Conversely, *S. aureus* with poorly cross-linked cell walls after sub-MIC antibiotic exposure may release a significant amount of toxins and other pathogenic factors that may exacerbate the host’s inflammatory response [3].

## Conclusion

The high biofilm production induced by sub-MICs of ampicillin, ceftriaxone, gentamicin, and norfloxacin has potential clinical relevance and may lead to chronic infections that are difficult to treat. More in vivo experiments are needed to determine whether sub-MIC levels of these antibiotics enhance biofilm formation in clinical settings, and further studies are required to determine the clinical significance of the morphological alterations in *S. aureus* caused by sub-MIC ceftriaxone.

## Data Availability

All data generated or analyzed during this study are included in this published article.

## Abbreviations

*ATCC*: American Type Culture Collection
*eDNA*: Extracellular DNA
*MRSA*: Methicillin-resistant *S. aureus*
*PBS*: Phosphate-buffered saline
*PIA*: Polysaccharide intercellular adhesion
*RT-PCR*: Real-time polymerase chain reaction
*S. aureus*: *Staphylococcus aureus*
*SCV*: Small colony variant
*SEM*: Scanning electron microscope
*Sub-MICs*: Sub-inhibitory concentrations
